# Complete mitochondrial genome of *Cerace xanthocosma* and its phylogenetic position in the family Tortricidae

**DOI:** 10.1080/23802359.2020.1791750

**Published:** 2020-07-20

**Authors:** Jian-Hua Ding, Yan Yang, Jun Li

**Affiliations:** School of Life Sciences, Huaibei Normal University, Huaibei, China

**Keywords:** Mitochondrial genome, *Cerace xanthocosma*

## Abstract

*Cerace xanthocosma* Diakonoff, 1950 belongs to the Tortricidae of Lepidoptera, distributed in China and Japan. Its mitochondrial genome is 15,344 bp in size, containing 37 genes (13 protein-coding genes, 2 ribosomal RNA genes, and 22 transfer RNA genes) and a non-coding A + T-rich region. In the 37 genes, 14 genes are located on the minority-strand (N-strand) with the remaining genes on the majority-strand (J-strand). The A + T-rich region has a poly-T stretch and a motif ATAGA. Phylogenetic analysis using Bayesian Inference method shows the sister relationship between Tortricinae and Olethreutinae with the placement of *C. xanthocosma* as the basal lineage of Tortricinae.

The species of Tortricidae has a common name “leafrollers” owing to its larval habit of shelter-building by folding or rolling leaves of the food plant (Brown et al. 2008), and there are more than 11,300 species of leafroller moths in the world (Gilligan et al. 2018). Currently, the Tortricidae is divided into three subfamilies: Tortricinae, Chlidanotinae, and Olethreutinae. The genus *Cerace* belongs to the Tortricinae, and *Cerace xanthocosma* Diakonoff, 1950 is distributed in China and Japan. As an important economic pest, the larvae of *C. xanthocosma* feed on many plants such as *Camellia*, *Symplocos*, *Acer*, and *Castanopsis* (Liu and Li 2002).

The mitochondrial genome (or mitogenome) is a typically double-stranded and circular DNA molecule containing 37 genes (13 protein-coding genes (or PCGs), 22 transfer RNA genes (or tRNAs), and 2 ribosomal RNA genes (or rRNAs)) and one non-coding A + T-rich region (Cameron 2014). Due to small size, abundance in cells, maternal inheritance, and high evolutionary rate, mitogenomes have been widely used to study molecular systematics, population genetics, and molecular evolution of insects (Shao et al. 2003; Ma et al. 2012; Nelson et al. 2012). More sequences of mitogenomes would help us understand the genomic characteristics and the phylogeny of Tortricidae. Consequently, we sequenced the complete mitogenome of *C. xanthocosma*.

The moths of *C. xanthocosma* were collected from Huangshan Mountains, Anhui Province, China (30°05′21′′ N, 118°08′48′′ E). The methods of DNA extraction, amplification, and sequencing were the same as those described in Li et al. (2018, 2019). The specimen (accession number is 20170526013) and the template DNA (accession number is 20170526013DNA) were respectively deposited in the Specimens Room and the Human and Animal Genetics Laboratory, School of Life Sciences, Huaibei Normal University, China.

The complete mitogenome of *C. xanthocosma* is 15,344 bp in size (the GenBank accession number is MT499230), which is just within the sequenced mitogenome length of leafrollers ranging from 15,224 bp of *Lobesia* sp. (GenBank accession number: KX621053) to 16,056 bp of *Eudemis porphyrana* Hübner, 1796/99 (GenBank accession number: MK820027). It consists of 40.7% A, 40.4% T, 11.4% C, and 7.5% G. Like other lepidopterans, the mitogenome of *C. xanthocosma* also has a typical set of 37 genes and a non-coding A + T-rich region (11,225 bp of 13 PCGs, 1462 bp of 22 tRNAs, 2170 bp of 2 rRNAs, and 383 bp of A + T-rich region). In the 37 genes, 4 PCGs (*nad5*, *nad4*, *nad4L*, and *nad1*), 8 tRNAs (*trnQ*, *trnC*, *trnY*, *trnF*, *trnH*, *trnP*, *trnL1*, and *trnV*), and 2 rRNAs are located on the minority-strand (N-strand) with the remaining 23 genes on the majority-strand (J-strand), which is identical to the other lepidopterans (Li et al. 2019; Ding et al. 2020). All PCGs start with ATN and stop with TAA codons except *cox1* which starts with CGA codon. For *cox1*, different species may have different start codons (e.g., in Isoptera it is ATT (Cameron and Whiting 2007) while in Orthoptera it is CCG (Fenn et al. 2007)), which is considered to be common across insects (Fenn et al. 2007).

The A + T-rich region was considered as the control region identified in insects including the origin sites for transcription and replication (Taanman 1999; Cameron and Whiting 2008). Same as the other lepidopterans, the A + T-rich region of *C. xanthocosma* mitogenome is also located between *rrnS* and *trnM* with a poly-T stretch and the “ATAGA” motif sequence. However, the size of this A + T-rich region (383 bp) is relatively small in lepidopterans, which is generally considered as the main reason leading the differences in the size of mitogenomes among insect species (Dai et al. 2018).

The sizes of 22 tRNAs of *C. xanthocosma* mitognome range from 62 to 71 bp, comprising 9.5% (1462 bp) of the complete mitogenome. The gene orientation and arrangement of tRNAs is *trnM*-*trnI*-*trnQ*, which is considered as a character derived from ancestral gene order *trnI*-*trnQ*-*trnM* (Boore 1999). Two rRNAs are 2170 bp in size. The larger one (*rrnL*) is located between *trnL1* and *trnV*, while the smaller is located between *trnV* and A + T-rich region. This arrangement is also the same as the other sequenced lepidopterans.

Up to now, 28 complete mitogenome sequences belonging to 2 subfamilies (Tortricinae and Olethreutinae) of Tortricidae have been verified in GenBank. Phylogenetic analysis based on these complete mitogenome sequences was performed using Bayesian Inference (BI) method. *Eogystia hippophaecola* Hua, Chou, Fang & Chen, 1990 (Lepidoptera: Cossidae) was used as the outgroup. The result shows the sister relationship between Tortricinae and Olethreutinae with the placement of *C. xanthocosma* as the basal lineage of Tortricinae (Figure 1). It is noticeable that *Acleris fimbriana* Thunberg & Becklin, 1791 (Tortricinae) was clustered as the basal lineage of the two subfamilies, which means that Tortricinae may not be a monophyletic group and needs more data for the further study. In addition, two sequences of *Choristoneura occidentalis* (MG948539 and MG948541) were not clustered together, which also needs further study.

**Figure 1.**
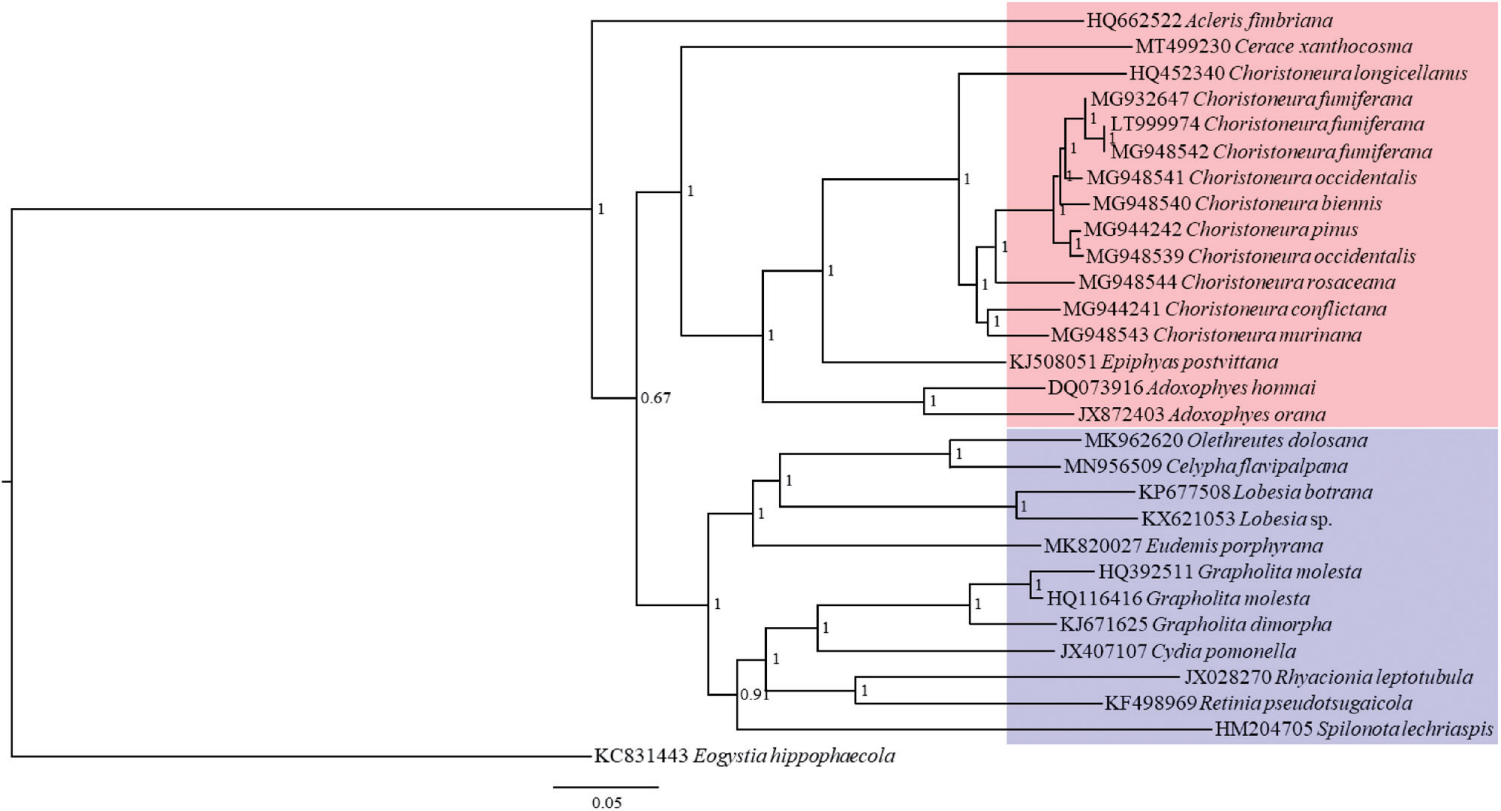
Phylogenetic tree for Tortricidae based on 28 mitogenome sequences of 14 genera with Bayesian Inference (BI) method. The numbers at the nodes mean the Bayesian posterior probability. The scale bar indicates the number of nucleotide substitutions per site in the sequence. GenBank accession numbers of mitogenome sequences are listed before the scientific names of species. The species with red background belong to Tortricinae, and blue belongs to Olethreutinae.

## Data Availability

The complete mitochondrial genome sequence and annotation of *Cerace xanthocosma* that support the findings of this study are openly available in Zenodo at https://doi.org/10.5281/zenodo.3913090
